# Large-Scale Distribution and Activity of Prokaryotes in Deep-Sea Surface Sediments of the Mediterranean Sea and the Adjacent Atlantic Ocean

**DOI:** 10.1371/journal.pone.0072996

**Published:** 2013-08-28

**Authors:** Donato Giovannelli, Massimiliano Molari, Giuseppe d’Errico, Elisa Baldrighi, Claudia Pala, Elena Manini

**Affiliations:** 1 Institute for Marine Science - ISMAR, National Research Council of Italy - CNR, Ancona, Italy; 2 Institute for Marine and Coastal Science - IMCS, Rutgers University, New Brunswick, New Jersey, United States of America; 3 Max Planck Institute for Marine Microbiology, Bremen, Germany; 4 Department of Bioscience, University of Parma, Parma, Italy; U. S. Salinity Lab, United States of America

## Abstract

The deep-sea represents a substantial portion of the biosphere and has a major influence on carbon cycling and global biogeochemistry. Benthic deep-sea prokaryotes have crucial roles in this ecosystem, with their recycling of organic matter from the photic zone. Despite this, little is known about the large-scale distribution of prokaryotes in the surface deep-sea sediments. To assess the influence of environmental and trophic variables on the large-scale distribution of prokaryotes, we investigated the prokaryotic assemblage composition (*Bacteria to Archaea* and *Euryarchaeota* *to Crenarchaeota* ratio) and activity in the surface deep-sea sediments of the Mediterranean Sea and the adjacent North Atlantic Ocean. Prokaryotic abundance and biomass did not vary significantly across the Mediterranean Sea; however, there were depth-related trends in all areas. The abundance of prokaryotes was positively correlated with the sedimentary concentration of protein, an indicator of the quality and bioavailability of organic matter. Moving eastwards, the *Bacteria* contribution to the total prokaryotes decreased, which appears to be linked to the more oligotrophic conditions of the Eastern Mediterranean basins. Despite the increased importance of *Archaea*, the contributions of Crenarchaeota Marine Group I to the total pool was relatively constant across the investigated stations, with the exception of Matapan-Vavilov Deep, in which Euryarchaeota Marine Group II dominated. Overall, our data suggest that deeper areas of the Mediterranean Sea share more similar communities with each other than with shallower sites. Freshness and quality of sedimentary organic matter were identified through Generalized Additive Model analysis as the major factors for describing the variation in the prokaryotic community structure and activity in the surface deep-sea sediments. Longitude was also important in explaining the observed variability, which suggests that the overlying water masses might have a critical role in shaping the benthic communities.

## Introduction

The deep-sea floor represents a substantial portion of the biosphere, as it covers approximately 65% of the Earth surface. It constitutes a dynamic environment that is linked to the upper water column processes [1], and it has a major influence in carbon cycling and global biogeochemistry [2–4]. Moreover, it has also become clear that the microbial processes that occur along the deep-sea floor are essential to sustain oceanic primary and secondary production [5,6].

Recent estimates indicate that at least 2.9 ×10^29^ prokaryotes reside in the first few meters of sediment depth [7]. Prokaryotes are key players in all ecosystems, and in the deep-sea they have a crucial role in recycling particulate and dissolved organic matter that sinks down from the photic zone [8]. Despite their importance, little is known about the large-scale distribution of prokaryotes in the surface deep-sea sediments [9,10], as most of the scientific literature has focused on the water column [11–14].

Even less is known on the larger scale of the ratio between the *Bacteria* and *Archaea* domains in the top sediment layers [15–17]. *Bacteria* dominance in surface sediments is generally accepted [15–20], and *Archaea* account for 5% to 30% of the total prokaryotic abundance [17]. This value increases with increasing sediment and water depth [11,17,21–23]. While the importance of *Bacteria* in biogeochemical cycles is well established [24–27], the role of *Archaea* in the functioning of marine systems is still poorly understood. *Archaea* have been regarded as organisms that inhabit extreme environments [28], although they are now known to be widespread throughout the oceans of the world [28–31], where they constitute a relevant fraction of the microbial community [22].

Previous studies have reported Crenarchaeota Marine Group I (MG-I) as the most abundant component of the *Archaea* population in oxygenated deep waters [22,25] and surface sediments [17,32–35], surpassing Euryarchaeota Marine Group-II (MG-II) abundance by *ca*. five-fold. *Euryachaeota* are an *Archaea* group that comprises the most extreme halophiles (e.g., genera *Halobacterium* , *Haloaredivivus*) and methanogens (e.g., genera *Methanococcus* , *Methanothermus* ), including also methanotrophs (e.g., *ANME-1 cluster*, *Methanosarcina* ) and thermophiles (e.g., *Thermococcus* ).

Prokaryotic distribution, abundance and community composition are controlled by environmental and trophic variables [17,36–40]. To date, only regional and local scale driving factors have been investigated, and although depth-related trends in prokaryotic abundance distribution have been reported [41–43], the enduring controlling factors of the variability of prokaryotic parameters (i.e., abundance, biomass, activity) appear to be the amount and availability of organic matter that settles to the seafloor [36–38,44–46]. In deep-sea sediments, the quantity and quality of organic matter is largely dependent upon seasonal deposition and burial of organic matter produced in the photic layer, as well as the complex biochemical transformations of the particles as they sink down the water column [47]. Thus, the quality and quantity of the organic carbon is considered to control the distribution of heterotrophic prokaryotes in marine sediments [46]. To this, we need to add the contribution of *in-situ* and local processes, such as dark CO_2_ fixation (i.e., autotrophic fixation of carbon in the absence of light [48]), the viral shunt [49], which diverts large quantities of organic matter back into the microbial loop, and the lateral inputs and stochastic events (deep-water currents, lateral advection and cascading [50]).

We present here the data collected in the course of five oceanographic cruises. We analyzed the influence of environmental and trophic variables on the large-scale distribution of prokaryotic assemblages and activity in the deep-sea surface sediments of the Mediterranean Sea and the adjacent Atlantic Ocean. We assumed during our analyses that on the large scale, different variables can come into play, and latitude and longitude might represent important forcing variables that hide the effects of local factors [9,12]. This implies that geographic position might have a strong influence on the functioning and contribution of *Archaea* and *Bacteria* to prokaryotic assemblages.

## Materials and Methods

### Sampling

**Figure 1:**
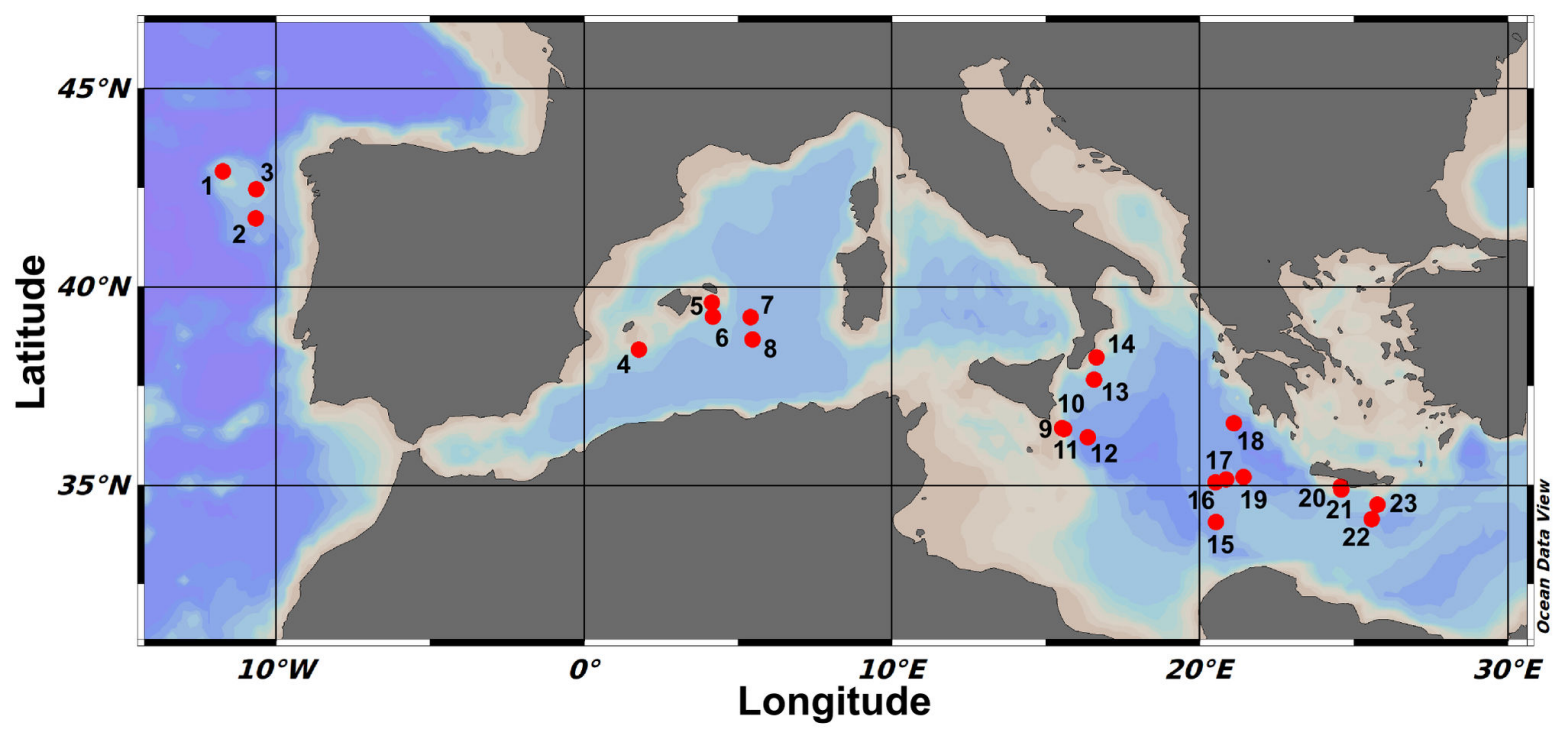
Map of the sampled stations across the Mediterranean Sea and North Atlantic Ocean. Numbers refer to the stations according to their longitude (see Tables 1, 2).

Sediments samples were collected during five oceanographic cruises in 2008 and 2009, as part of the EU-funded project ESF-EuroDeep BIOFUN (Biodiversity and Ecosystem functioning in contrasting southern European deep-sea environments). North Atlantic stations were sampled in October 2008, while Mediterranean Sea stations were sampled between June 2008 and May/June 2009 (stations 15, 16, 17, 18, 19, 20 and 21 in June 2008; stations 4, 7, 8, 9, 10, 11, 12, 13, 14, 22 and 23 in between May and June 2009), with the exception of stations 5 and 6 sampled in November 2009. For details on the sampling locations and depths, see Table 1 and Figure 1. No specific permission were required for locations/activities described in this study, as most of the activities were carried out in international waters, except in Greek waters, where appropriate permission was obtained by the Ministry of Foreign Affairs of the Hellenic Republic. All the sampling and field studies did not involve endangered or protected species. Undisturbed sediments were collected by box-corer (n = 3) and sub-sampled on board, with the collection and processing of the top 1 cm of sediments. Aliquots were immediately frozen at -20 °C for the determination of the organic matter composition. Sediment sub-samples were directly analyzed for heterotrophic production, and replicates of about 1 ml wet sediment were fixed using buffered formaldehyde (final concentration, 2%; in sterile and filtered seawater), and stored at 4 °C until processed for total prokaryotic abundance and biomass determination [51,52]. To investigate the prokaryotic assemblage composition in term of *Bacteria*/*Archaea* and *Euryarchaeota* */Crenarchaeota* abundance, sediment sub-samples (0.5 g) were fixed in 4.5 ml formaldehyde (final concentration, 2%; in phosphate-buffered saline [PBS], pH 7.4) for 1 h at room temperature. The fixed samples were then washed three times with PBS (centrifugation of 10,000× *g* for 5 min between washes), and then stored in PBS/ethanol (1:1; v/v) at -20 °C, until further processing [16].

**Table 1:** Details of the sampling stations.

**Station**	**Cruise**	**Depth**	**Area**	**Latitude**	**Longitude**	**Bottom temperature**	**SPP**
		**(m)**		**(°N)**	**(°E)**	**(°C)**	**(mgC m^-2^ d^-1^)**
1	Pelagia 08	1200	N	42.9118	-11.7525	9.6	480.9
2	Pelagia 08	3000	N	41.7285	-10.6835	2.7	523.3
3	Pelagia 08	2000	N	42.4607	-10.6547	4.0	546.8
4	Trans-Med 09	1200	W	38.4203	1.7704	13.0	434.6
5	Pelagia 09	1200	W	39.5997	4.1454	13.0	352.5
6	Pelagia 09	2000	W	39.2498	4.1667	13.2	315.8
7	Pelagia 09	3000	W	39.2363	5.4019	13.3	432.5
8	Trans-Med 09	3000	W	38.6776	5.4647	13.3	430.2
9	Biofun 09	1200	C	36.4327	15.5174	13.7	650.0
10	Biofun 09	2000	C	36.4222	15.5458	13.7	614.7
11	Biofun 09	2000	C	36.4168	15.5836	13.8	628.4
12	Trans-Med 09	3000	C	36.1987	16.3526	13.9	353.6
13	Trans-Med 09	2000	C	37.6539	16.5591	13.8	319.4
14	Trans-Med 09	1200	C	38.2237	16.6298	13.7	384.7
15	Biofun 08	3000	C	35.0732	20.5045	14.7	315.8
16	Biofun 08	3000	C	35.0682	20.5075	14.7	278.6
17	Biofun 08	3000	C	35.1388	20.8482	14.7	309.9
18	Biofun 08	5000	C	36.5597	21.0984	14.7	325.2
19	Biofun 08	3000	C	35.1972	21.4075	14.7	341.3
20	Biofun 08	1200	E	34.9539	24.5709	14.7	312.8
21	Biofun 08	3000	E	34.8833	24.5875	14.7	308.1
22	Trans-Med 09	3000	E	34.1459	25.5696	13.9	268.4
23	Trans-Med 09	1200	E	34.5061	25.7590	13.9	285.0

SPP, surface primary production

### Sedimentary organic matter

Total protein (PRT), carbohydrate (CHO), lipid (LIP), chlorophyll-a and phaeopigments were determined according to [53]. Concentrations were calculated using standard curves, and normalized to sediment dry weight after desiccation (60 °C, 24 h). Protein, carbohydrate and lipid concentrations were converted into C equivalents using the conversion factors of 0.49, 0.40 and 0.75 µgC µg^-1^, respectively [54]. Biopolymeric organic C (BPC) was calculated as the sum of the C equivalents of protein, carbohydrate and lipid, and this was used as a proxy for the available trophic resources [55]. Chloroplastic pigment equivalents (CPE) are defined here as the sum of the chlorophyll-a and phaeopigment concentrations.

### Surface primary production

In order to account for possible differences in sampling season within our dataset (North Atlantic stations *vs* Mediterranean stations), we included estimates of surface primary production at the time of sampling. Data were obtained from the Ocean Productivity database (url: http://www.science.oregonstate.edu/ocean.productivity/), for the sampling area and the period, and using the vertically generalized production model as described in [56]. This model estimates depth-integrated net primary production (mgC m^-2^ d^-1^) based on surface chlorophyll (mg m^-3^), surface photosynthetic active radiation, and sea-surface temperature. Despite the effort to include all relevant variables in our analysis in order to exclude seasonal and stochastic influences on our observations, the potential role of sampling season on the difference measured between the North Atlantic and Mediterranean stations cannot be ruled out.

### Total prokaryotic numbers and biomass

Total prokaryotic counts (TPN) were performed using an acridine orange staining technique [57]. Briefly, tetrasodium pyrophosphate was added to 0.5 g sub-samples, which were incubated for 15 min in the dark before sonication. The samples were then stained with acridine orange (final concentration, 0.025%), filtered on 0.2 µm pore-size polycarbonate filters under low vacuum, and analyzed as described by 58, using epifluorescence microscopy (magnification, 1,000×). Prokaryotic biomass (PBM) was estimated using a micrometer ocular, assigning prokaryotic cells into different size classes based on their maximum length and width [58]. These were converted into biovolumes assuming an average C content of 310 fgC µm^-3^ [58]. TPN and PBM were normalized to sediment dry weight after desiccation (24 h at 60 °C).

### Heterotrophic production

Heterotrophic production (HCP) was measured using [^3^H]-leucine incorporation, following a procedure described by 57. Briefly, a saturated aqueous solution of [^3^H]-leucine (specific activity 67-73 Ci mmol^-1^, final concentration, 3 µCi) was added to sediment sub-samples (0.2 ml), which were incubated for 1 h in the dark at their *in-situ* temperature. Three sediment blanks were run in parallel, adding ethanol immediately before the [^3^H]-leucine addition. After the incubation, prokaryotic C incorporation was stopped by adding 1.7 ml 80% ethanol, and the samples were stored at 4 °C until processed in the laboratory. The samples were then washed three times with 80% ethanol, and immediately filtered under low vacuum (<100 mm Hg) on 0.2 µm pore-size Nucleopore filters. The filters were washed four times with 2 ml 5% trichloroacetic acid, transferred into pyrex test tubes, treated with 2 N NaOH, and incubated at 100 °C for 2 h. After centrifugation to separate the sediment residue (800× *g*, 10 min), 1 ml supernatant was transferred to scintillation vials containing scintillation fluid (Hionic Fluor; Packard Bioscience). The incorporated radioactivity was measured by determining the [^3^H]-cpm in a liquid scintillation counter (Packard Tri-Carb 300). [^3^H]-leucine incorporation was converted into C produced by the heterotrophic prokaryotes according to [59], using 1.55 kgC mol^-1^ leucine. The data were normalized to sediment dry weight after desiccation (60 °C, 24 h). The cell specific activities were calculated as HCP/active cells (fgC cell^-1^ h^-1^).

### Catalyzed reporter deposition - fluorescence in-situ hybridization (CARD-FISH)

To investigate the contribution of *Bacteria* and *Archaea* to total prokaryotes and the contribution of *Euryarchaeota* and *Crenarchaeota* to total *Archaea*, fluorescence in-situ hybridization (FISH) was used with rRNA-targeted oligonucleotide probes and signal amplification (CARD; CAtalyzed Reporter Deposition), as described previously [16,60,61]. The oligonucleotide probes used were EUB338-mix (EUB338, 5’-GCT GCC TCC CGT AGG AGT-3’, EUB338-II, 5’-GCA GCC ACC CGT AGG TGT-3’, and EUB338-III, 5’-GCT GCC ACC CGT AGG TGT-3’), which targeted total *Bacteria* [62,63], ARCH915 (5’-GTG CTC CCC CGC CAA TTC CT-3’), which targeted total *Archaea* [64], CREN537 (5’-TGA CCA CTT GAG GTG CTG-3’) which targeted Crenarchaeota Marine Group I [11], EURY806 (5’-CAC AGC GTT TAC ACC TAG-3’) which targeted Euryarchaeota Marine Group II [11], and NON338 (5’-ACT CCT ACG GGA GGC AGC-3’) as the negative control [65]. Briefly, bacterial cell-wall permeabilization was achieved by incubating the filters in lysozyme solution (10 mg ml^-1^) at 37 °C for 1 h; archaeal permeabilization was achieved by incubating the filters in proteinase K (0.4 mU ml^-1^), as described by 61. After probe hybridization and washing, the signals were amplified by incubation with tyramide-Cy3. The filters were analyzed by epifluorescence microscopy (magnification, 1,000×) with an appropriate filter set for Cy3 fluorescence. The prokaryotic assemblages dominance by *Bacteria* or *Archaea* was calculated as the Bacteria to Archaea ratio (BAR), which has a value of 1 for a population that consists of 50% of each domain, in order to give a synoptic view of the relative abundance. Similarly, the *Euryarchaeota* to *Crenarchaeota* ratio (ECR) was calculated on the same principle [17].

### Statistical analyses

The stations were grouped into areas on the basis of their geographic positions: North Atlantic Ocean (N, 3 stations), and West (W, 5 stations), Central (C, 11 stations) and East (E, 4 stations) Mediterranean Sea, and used as a factor in the following analyses. Depth was also used as a factor, with 4 levels (1,200, 2,000, 3,000, 5,000 m). Map plots were drawn using Ocean Data View [66]. All of the statistical analyses were performed using the R software [67].

The samples were investigated for differences in measured variables among the stations, sampling areas and depths, using ANOVA. Where ANOVA assumptions were rejected, a more conservative level of *p* was chosen [68]. In cases of significant differences, a HSD Tukey post-hoc test was performed.

To investigate the presence of trends in our sampling design, multivariate ordination analysis was performed. Non-metric multi-dimensional scaling (nMDS) is an ordination technique in which variables describing a multidimensional space are scaled based on their similarity on a two-dimension plot, to maximize the distances among the points [69]. nMDS plots have no axis scales or meaningful absolute units for the axes, the relative distances between plotted points being the only meaningful result. This analysis was performed using the nMDS function from the Vegan R-package [70] on the descriptors of the prokaryotic assemblages (TPN, PBM, BAR, ECR, HCP). The environmental (LAT, LONG, Depth, Temperature) and trophic (BPC, PRT, LIP, CHO, CPE) variables were superimposed with the ordisurf function of the Vegan package, to fit generalized additive model (GAM) surfaces to the ordination. Using this approach the relationships between environmental and trophic variables and the observed prokaryotic assemblages ordination were explored for linearity. Only significant relationships were plotted over the nMDS ordination. In order to investigate depth-related trends in the analysis of basin-wide differences in prokaryotic assemblages, the nMDS analysis was repeated splitting the data according to sampling depth.

To understand the importance of the environmental and trophic variables in describing the prokaryotic assemblages composition (expressed as BAR and ECR) and functioning (HCP) variations across the Mediterranean Sea, a GAM analysis was performed using the mgcv R-package [71]. GAMs are an advancement of generalized linear models, in which for every parameter added to the model, a spline function is applied, to perform a smooth (i.e., non-linear) fitting. Using this approach is possible to identify hidden relationships among variables that show only weak linear correlation. The added complexity in the resulting models is balanced by the increased accuracy in the model prediction power. Models are built through successive additions of describing variables. The model selection was carried out using the Akaike Information Criterion [72], the generalized cross-validation score [71] and increased accuracy of the model. To our knowledge this is the first time GAMs have been applied to microbial ecology in marine environments.

## Results

### Trophic variables

**Figure 2:**
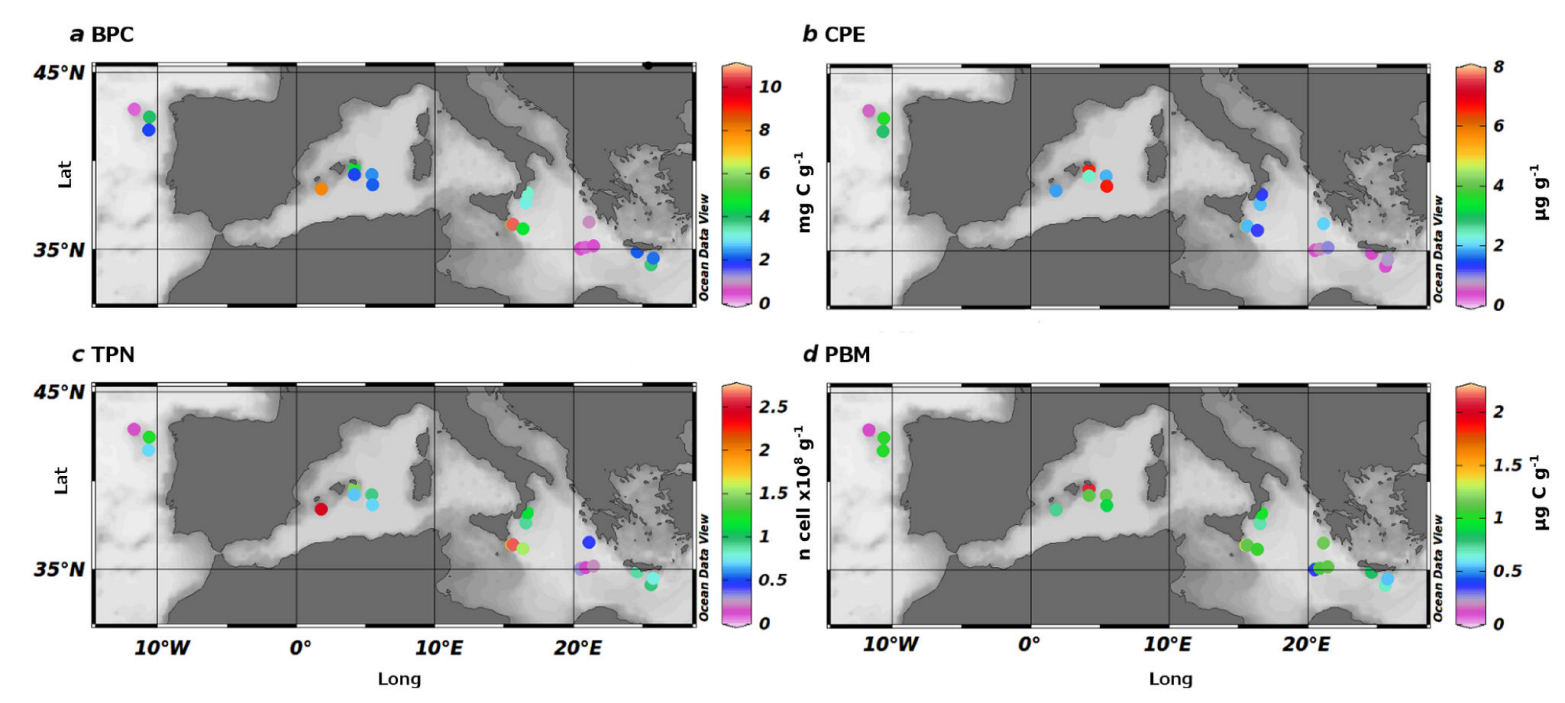
Maps showing the trophic and prokaryotic variables across the Mediterranean Sea and North Atlantic Ocean. (a) Biopolymeric carbon (BPC). (b) Chloroplastic pigment equivalent (CPE). (c) Total prokaryotic counts (TPN). (d) Prokaryotic biomass (PBM).

**Figure 3:**
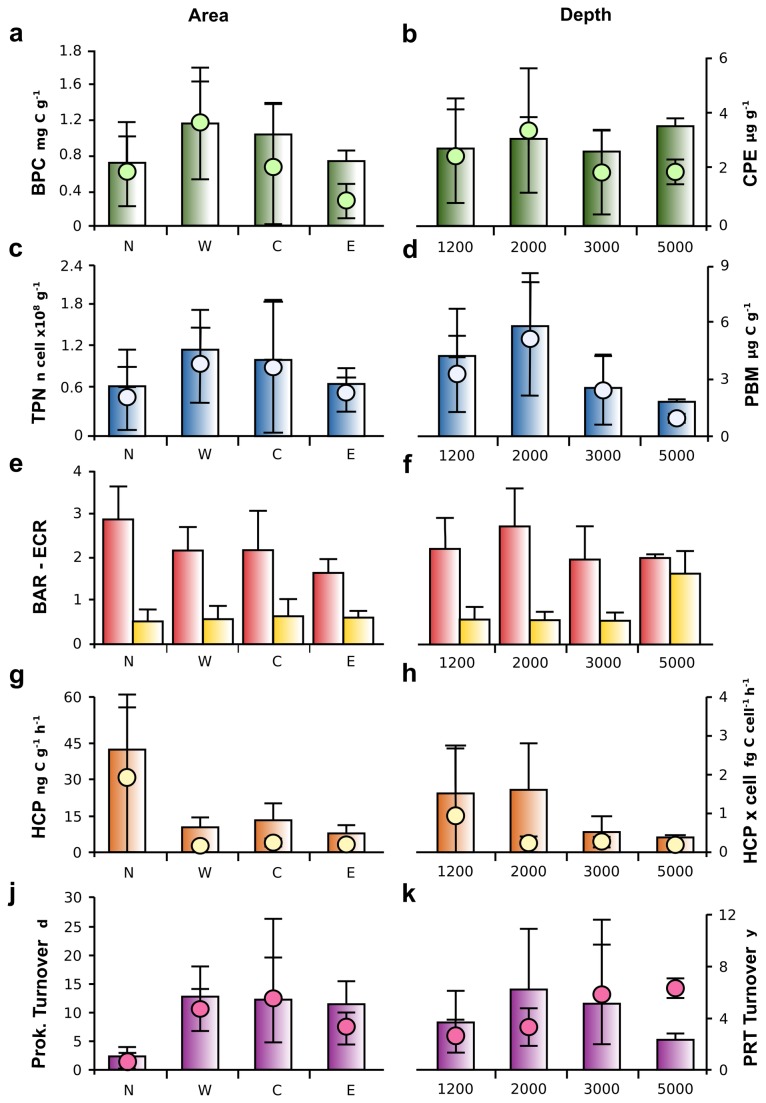
Measured variables across the sampling areas and sampling depths. Left axes, barplots; right axes, dot plots. (a, b) Biopolymeric carbon (BPC) and chloroplastic pigment equivalent (CPE) according to area (a) and depth (b). (c, d) Total prokaryotic counts (TPN) and prokaryotic biomass (PBM) according to area (c) and depth (d). (e, f) Bacteria to Archaea ratio (BAR) and *Euryarchaeota* to *Crenarchaeota* ratio (ECR) according to area (e) and depth (f). (g, h) Heterotrophic production (HCP) and cell specific activity (HCP x cell) calculated on the active cell according to area (g) and depth (h). (j, k) Prokaryotic turnover time and Protein turnover time according to area (j) and depth (k). Means and standard deviations are reported. N, North stations; W, West stations; C, Central stations; E, East stations.

The BPC content of the surface sediment and the CPE are plotted over the sampling area in Figure 2a, b, with the reported averages for area and depth in Figure 3a, b, respectively. On average, the West and Central Mediterranean accounted for the higher amounts of BPC in sediment (1.16 ±0.48 and 1.03 ±0.35 mgC g^-1^, respectively), with the BPC quantity evenly distributed across the depths (Figure 3b). CHO was the dominant category in organic matter, constituting, on average, 50% of the total organic matter, with PRT following with an average contribution of 37% (Table 2). The CPE followed a similar trend, with higher values in the West and Central Mediterranean (3.75 ±2.05 and 2.21 ±2.25 µg g^-1^, respectively), while showing a higher variability along the depth transects, with average values higher at 2,000 m (3.46 ±2.61 µg g^-1^; Figure 3b). The CPE showed good correlation with surface primary production (SPP; Pearson moment correlation, n = 69, r = 0.51, p <0.05).

**Table 2:** Sedimentary organic matter content in the sampled stations.

**Station**	**Depth**	**Area**	**PRT**	**CHO**	**LIP**
	**(m)**		**(mg g^-1^ [±sd])**	**(mg g^-1^ [±sd])**	**(mg g^-1^ [±sd])**
1	1200	N	0.042 [0.025]	0.223 [0.013]	0.018 [0.003]
2	3000	N	0.282 [0.035]	1.624 [0.118]	0.272 [0.042]
3	2000	N	0.766 [0.019]	1.350 [0.180]	0.145 [0.038]
4	1200	W	0.745 [0.092]	0.517 [0.126]	0.201 [0.001]
5	1200	W	1.537 [0.201]	2.318 [0.187]	0.452 [0.137]
6	2000	W	0.907 [0.177]	1.190 [0.158]	0.180 [0.033]
7	3000	W	0.966 [0.286]	1.063 [0.284]	0.295 [0.025]
8	3000	W	0.701 [0.076]	1.058 [0.134]	0.156 [0.032]
9	1200	C	1.671 [0.279]	0.455 [0.080]	0.743 [0.114]
10	2000	C	1.581 [0.196]	0.542 [0.029]	0.557 [0.022]
11	2000	C	1.267 [0.066]	0.604 [0.178]	0.326 [0.085]
12	3000	C	1.304 [0.400]	0.554 [0.204]	0.349 [0.131]
13	2000	C	0.521 [0.080]	0.736 [0.058]	0.171 [0.017]
14	1200	C	0.788 [0.228]	0.975 [0.044]	0.093 [0.036]
15	3000	C	0.493 [0.069]	1.325 [0.061]	0.244 [0.024]
16	3000	C	0.450 [0.098]	0.186 [0.010]	0.009 [0.000]
17	3000	C	0.460 [0.023]	1.491 [0.169]	0.253 [0.006]
18	5000	C	0.660 [0.127]	1.155 [0.066]	0.597 [0.083]
19	3000	C	0.530 [0.025]	1.506 [0.039]	0.296 [0.021]
20	1200	E	0.357 [0.116]	1.367 [0.063]	0.051 [0.001]
21	3000	E	0.442 [0.070]	1.094 [0.057]	0.286 [0.029]
22	3000	E	0.547 [0.153]	0.871 [0.107]	0.099 [0.011]
23	1200	E	0.345 [0.053]	0.915 [0.242]	0.046 [0.013]

PRT, total protein; CHO, total carbohydrate; LIP, total lipid

### Prokaryotic abundance and biomass

The TPN and PBM distributions along the Mediterranean Sea are shown in Figure 2c, d. No clear longitudinal gradients were present, and there were higher values of prokaryotic abundance at station 11, 2,000 m depth in the Central Mediterranean basin, with 2.61 ±0.01 ×10^8^ cell g^-1^. Despite the lack of significant trends, the TPN varied significantly across the areas, with higher values in the West and Central Mediterranean Sea (Figure 3c, Table 3; ANOVA, p <0.05). The minimum TPN of 1.32 ±0.21 ×10^7^ cell g^-1^ was found in the North Atlantic, at 1,200 m (station 1). Significant trends were also seen for the depth factor, with maximum values at 2,000 m and minimum at 5,000 m (Figure 3d; ANOVA, p <0.01). The interaction between area and depth revealed major variations across areas at 1,200 m, while these differences were absent at 2,000 and 3,000 m in depth (HSD-Tukey *post-hoc* test, p <0.05).

**Table 3:** Results of the ANOVA analyses on the prokaryotic variables.

**Variable**	**Area**				**Depth**				**Area × depth**		
	**df**	**F**	**P**		**df**	**F**	**P**		**df**	**F**	**P**
**TPN**	3	3.17	ns		3	11.56	***		5	6.96	**
**PBM**	3	2.82	ns		3	10.79	***		5	4.33	**
**HCP**	3	60.92	***		3	23.64	***		5	14.25	***
**BAR**	3	4.71	**		3	2.37	ns		5	1.15	ns
**ECR**	3	0.69	ns		3	17.82	***		5	3.89	**

df, degree of freedom; F, F test; P, probability level (***p <0.001, **p <0.01, ns, not significant);

TPN, total prokaryotic counts; PBM, prokaryotic biomass; HCP, heterotrophic production; BAR, *Bacteria* to *Archaea* ratio; ECR, *Euryarchaeota* to *Crenarchaeota* ratio

As seen in Figure 2d and Table 3, the PBM did not correlate with the TPN, and there were no significant differences across the areas (Figure 3c, Table 3), while the depth-related trends were consistent with prokaryote abundance (Figure 3c, Table 3; ANOVA, p <0.001). Once again, the highest values of the PBM were at station 11, with 9.83 ±0.68 µgC g^-1^. A significant interaction was found between area and depth (ANOVA, p <0.05; Table 3).

### Prokaryotic assemblages

The total number of prokaryotes counted using CARD-FISH accounted for an average of *ca.* 91% of the total prokaryotes counted using acridine orange, with minimum values at station 12 in the West Mediterranean at 1,200 m in depth of 60% ±7%, and maximum of 100% ±19% for station 20 at 1,200 m in depth in the East Mediterranean Sea. CARD-FISH analysis revealed a dominance of *Bacteria* over *Archaea* in all of investigated sediments (on average, BAR 2.2 ±0.83), with exception of station 17 in the Central Mediterranean (3,000 m in depth), where *Archaea* were the dominant prokaryotic domain (BAR 0.88 ±0.06). Higher values of BAR were detected in station 19 in the Central Mediterranean (3.67 ±0.56; 3,000 m in depth), followed by station 11 (3.60 ±0.75; Central Mediterranean; 2,000 m in depth) and station 1 (3.14 ±0.84; North Atlantic; 1,200 m in depth). The BAR varied significantly between areas (ANOVA, p <0.01; Table 3; Figure 3e), but not with depth (Figure 3f). There were no interactions between area and depth. The North Atlantic and East Mediterranean Sea were significantly different (Figure 3e, Table 3; HSD-Tukey *post-hoc* test, p <0.01), while the West and Central Mediterranean were a blend of the two values.

The dominance by *Euryarchaeota* or *Crenarchaeota* in the Archaea domain was described using the *Euryarchaeota* to *Crenarchaeota* ratio (ECR). The ECR was constant among the areas, with an overall mean of 0.57 ±0.35 (Figure 3e). *Crenarchaeota* MG-I dominated the Archaea community in all of the investigated stations, with the exception of station 18 in the Central Mediterranean Sea (1.64 ±0.58; Matapan-Vavilov Deep; 5,137 m in depth). Influenced by these data, the ECR changed significantly across the depths (ANOVA, p <0.001; Table 3, Figure 3f), with 5,000 m being different from all of the other depths (p <0.001; HSD-Tukey *post-hoc* test), and had no other statistically significant differences between 1,200 m, 2,000 m and 3,000 m in depth (Table 3).

### Prokaryotic activity in surface sediments

The HCP in the surface sediments ranged from 3.83 ±0.84 to 54.81 ±0.85 ngC g^-1^ h^-1^ (station 3, North Atlantic, 2,000 m in depth, and station 17, Central Mediterranean, at 3,000 m in depth, respectively), with an overall average of 16.32 ±16.20 ngC g^-1^ h^-1^. The HCP showed significant changes across the areas (ANOVA, p <0.001; Table 3, Figure 3g), with the North Atlantic surpassing other areas by *ca* 3-fold (HSD-Tukey *post-hoc* test, p <0.001; Figure 3g). Significant differences were also seen across the depths (ANOVA, p <0.001; Table 3, Figure 3h), with higher values at 1,200 m and 2,000 m in depth (23.92 ±19.76 and 26.14 ±18.09 ngC g^-1^ h^-1^, respectively) than at 3,000 m and 5,000 m in depth (8.3 ±5.84 and 6.01 ±1.03 ngC g^-1^ h^-1^, respectively). Cell specific activities were low for all investigated areas and depths, with the exception of North Atlantic stations, where they reached a maximum of 4.71 ±0.3 fgC cell^-1^ h^-1^ (Figure 3g, h). Prokaryotic turnover time, calculated as PBM/HCP, was on average 10.4 ±11.1 days with a minimum of 0.15 ±0.06 days in the North Atlantic at 1,200 m (station 1) and a maximum of 48.3 ±17.5 days in the central Mediterranean at 3,000 m (station 12). Statistically significant differences were present between areas (ANOVA, p<0.05; Figure 3j, k), with the North Atlantic showing prokaryotic turnover time three-fold lower on average than the Mediterranean stations.

The PRT concentrations in the sediments were converted into carbon equivalents and protein turnover time was calculated in the sediment, assuming steady-state conditions (i.e., no input of fresh protein to the system), using the following formula: C-PRT/HCP (years; Figure 3j, k). The lowest protein turnover time of 0.05 ±0.03 years was detected at station 1 (North Atlantic; 1,200 m in depth), while the maximum turnover of 13.7 ±5.9 years was for station 12 (Central Mediterranean; 3,000 m in depth). A protein turnover of 6.1 ±0.2 years was found for station 18, at 5,000 m in depth in the Matapan-Vavilov Deep.

### Relationship between environmental factors and whole prokaryotic community

**Figure 4:**
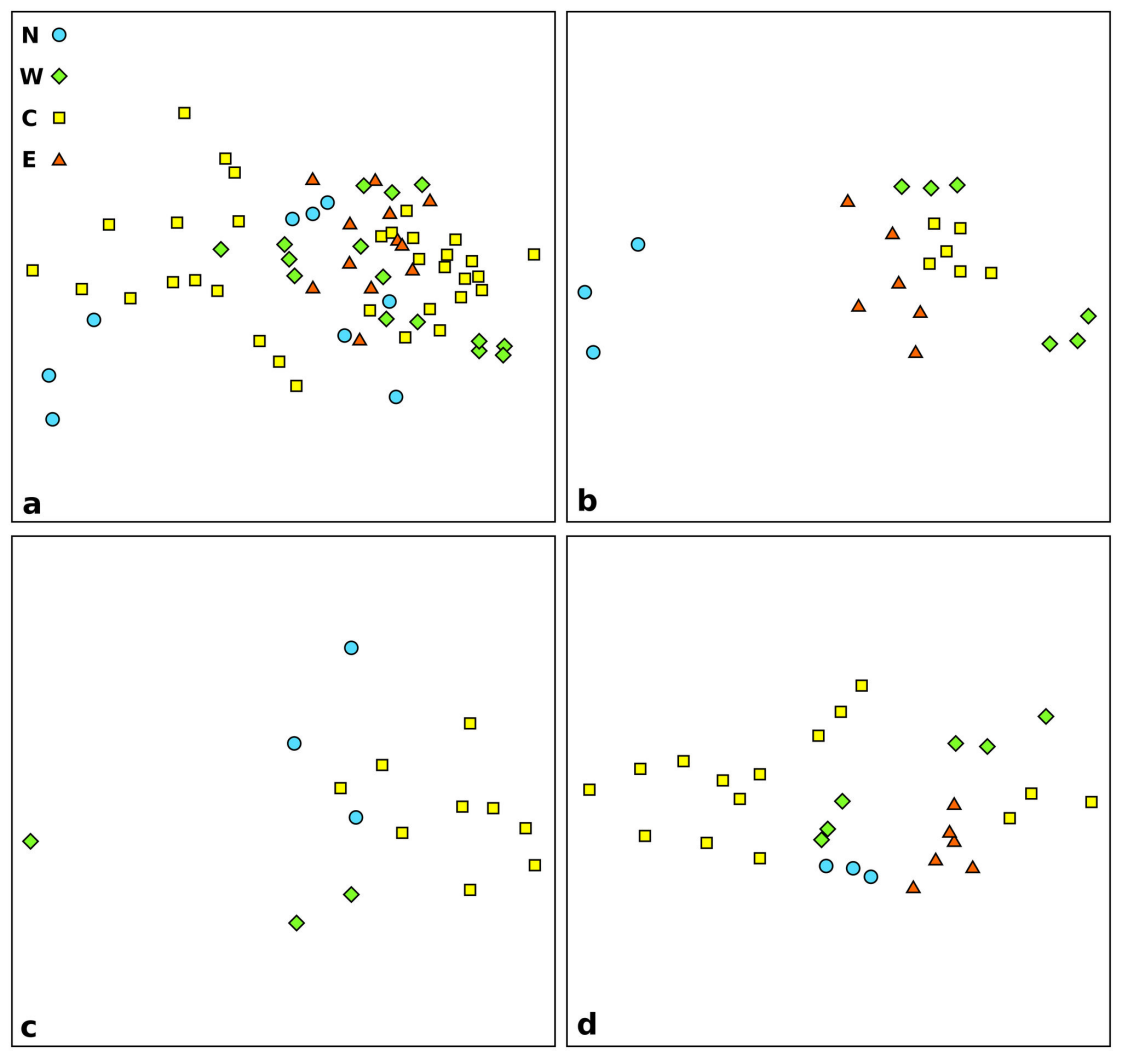
Non-metric multi-dimensional scaling analysis based on the biological descriptors of the prokaryotic community. The plots show all sampled stations (a), and those at 1,200 m (b), 2,000 m (c) and 3,000 m (d) in depth. N, North stations; W, West stations; C, Central stations; E, East stations.

To investigate the role of trophic and environmental factors on the distribution, activity and *Bacteria* to *Archaea* and *Euryarchaeota* to *Crenarchaeota* ratio, we performed non-metric multi-dimensional scaling analysis (nMDS). We plotted the nMDS of our whole dataset using prokaryotic variables (TPN, PBM, BAR, ECR and HCP) to describe the community in each sampled station (Figure 4a). The nMDS revealed a strong distribution of the stations along dimension 1, with a preponderant role of longitude in the ordination. The prokaryotic communities of stations from the East Mediterranean appeared to be very similar, clustering together in Figure 4a. The Central Mediterranean stations had the more diverse community, as these are interspersed with all of the other samples. To investigate depth-related effects, we repeated the nMDS analysis by separating the three main depths (Figure 4b–d). At 1,200 m in depth, the stations clustered together in discrete groups, based on the area, with the only exception of the West Mediterranean stations (Figure 4b). At 2,000 m (Figure 4c) and at 3,000 m (Figure 4d) the points were more interspersed.

**Figure 5:**
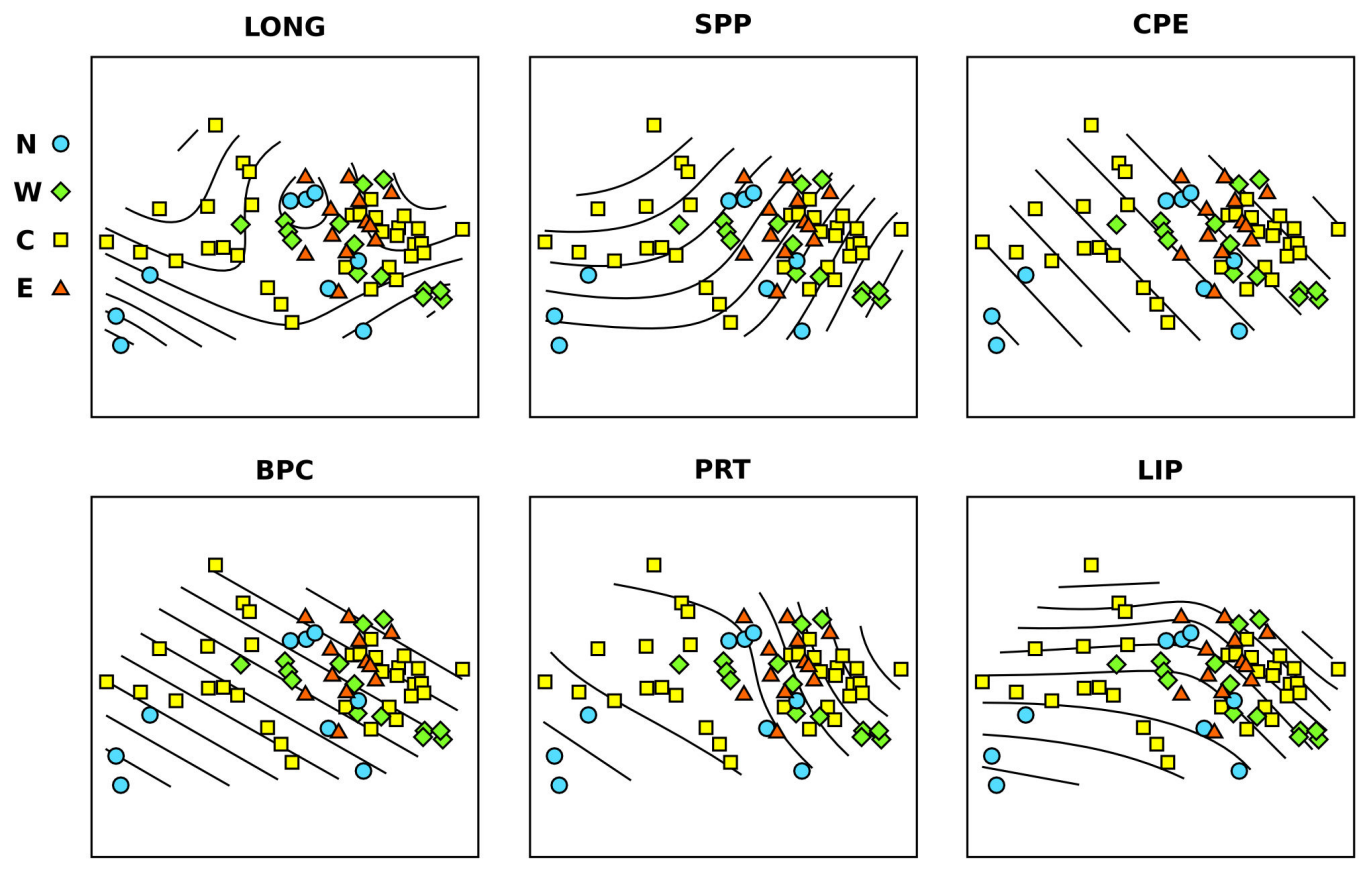
Non-metric multi-dimensional scaling analysis based on the biological descriptors of the prokaryotic community, with the GAM fitted surfaces. The plots show all of the sampled stations at all of the depths. The surface lines in each plot represent the GAM fitting of the significant environmental or trophic variables explaining the observed ordination in the plots. LONG, longitude; SPP, surface primary production; CPE, chloroplastic pigment equivalent; PRT, total protein; LIP, total lipid; N, North stations; W, West stations; C, Central stations; E, East stations.

Using GAM analysis to force our trophic and environmental factors on the nMDS ordination, we found that the trophic variables were the major driving factor in describing the differences in the prokaryotic communities between all of the sampled stations (Figure 5). Notably, LONG and SPP had relevant effects in separating the points in the ordination in a non-linear way (Figure 5). The same results were found for PRT and LIP, while the BPC and CPE surface fitted the ordination with a linear relationship.

### Generalized additive models

**Figure 6:**
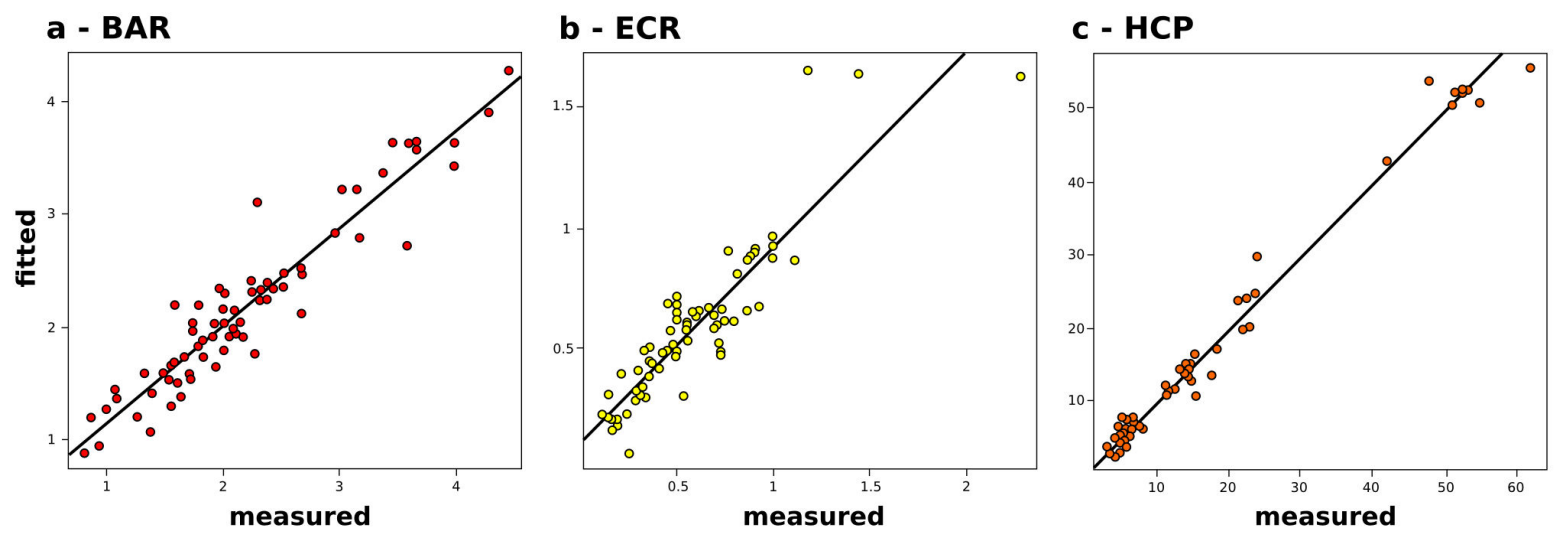
Linear regressions between the measured and GAM fitted values for prokaryotic variables. (a) Bacteria to Archaea ratio (BAR). (b) *Euryarchaeota* to *Crenarchaeota* ratio (ECR). (c) Heterotrophic production (HCP). The Pearson moment correlation coefficients are, respectively: 0.948 (df = 12.3, n = 69), 0.902 (df = 20.2, n = 69) and 0.972 (df = 27.5, n = 60).

GAM analysis was further used to identify the trophic and environmental factors that explained the BAR, ECR and HCP variations among the sampled stations (Figure 6). The BAR variation across our dataset was best explained by a combination of environmental (LAT and LONG) and trophic (SPP, CPE and PRT) variables. Interaction effects between LAT and LONG, and SPP and CPE were identified, and PRT was fitted using a spline function. The resulting model was able to predict 89.9% of the BAR variance across the sampled stations (df = 12.3). When the other trophic variables (e.g., BPC, PRT/CHO ratio) were added to the model, they only marginally increased its accuracy (i.e., BPC increased by only 1.4% of the variance prediction), although they significantly increased the complexity of the model. The interaction of SPP and CPE, together with the PRT, explained more than 60% of the variance, with the rest explained by the interaction of the LAT and LONG terms.

The ECR variations were explained by the interactions of LAT and LONG, and BAR and depth factor. This is influenced by the presence of a significantly different composition of the *Archaea* for station 18, at 5,000 m in depth, driving the effect of depth in explaining our ECR dataset. The BAR and depth were fitted as linear variables, and the overall model predicted 81.3% of the observed variance (df = 20.2).

The HCP across our dataset was best explained by the abundance of *Bacteria*, the interactions between SPP and CPE, the PRT content, and LONG. All of the variables were fitted as non-linear, resulting in higher complexity when compared to the previous GAM (df = 27.5). The resulting model explained 98.5% of the observed HCP variance in our dataset.

## Discussion

### Trends in prokaryotic abundance, biomass, assemblage composition and activity

It has been widely demonstrated than the prokaryotic abundance and biomass decrease with increasing depth, reaching their *minima* in the bathypelagic waters [73,74]. These decreases in abundance and biomass reported for the water columns have never been described on a large scale for surface sediments collected at different depths [75]. The Mediterranean Sea is well known for its peculiar characteristics: high deep-water temperature and homeothermy (*ca* 13 °C), fast deep-water turnover (in the order of 11 to 100 years [76]) and a strong decreasing trophic gradient moving from the Western Basin to the Eastern Basin [77]. Despite those differences with other basins, the prokaryotic community in the deep-water of the Mediterranean Sea follows a similar trend to those reported for other oceans [11,22,23,78,79], with a marked decrease in the abundance and biomass of prokaryotes, an increase in the abundance of *Archaea* with increasing water depth [23], and a decrease in prokaryotic heterotrophic production [79].

We investigated for the first time the abundance, assemblage composition and activity of prokaryotic communities in the surface deep-sea sediments of the Mediterranean Sea and North Atlantic Ocean as a large-scale survey. Our results show that the TPN does not display differences across the sampled areas (Figure 3c); however, it does show a clear trend with depth (Figure 3d), as a bell shaped distribution with maxima at 2,000 m in depth, as already described for other deep-sea fauna [75,80]. A similar trend was seen for the PBM (Figure 3c, d). The longitudinal trend appears to be related to the different trophic conditions present in the Mediterranean Sea, with higher TPN and PBM values in the Western Basin and lower values in the more oligotrophic Eastern Basin. Both of these results were well correlated with trophic resources. Using the PRT sedimentary concentration as a proxy for the bioavailability and freshness of the organic matter [81], we found a correlation between TPN and PRT (Pearson moment correlation, r = 0.70, n = 69, p <0.001) and Active cells and PRT (Pearson moment correlation, r = 0.67, n = 69, p <0.001), which suggests that the deep-sea Mediterranean surface sediments community is dominated by heterotrophic processes. This is supported by our measurements of HCP, which are high compared to other measurements made in surface sediments at similar depths [49,82], and which positively correlate with the PBM and active cells for the Mediterranean stations (Pearson moment correlation, r = 0.50, n = 60, p <0.001, and r = 0.54, n = 60, p <0.001, respectively). However, when we calculated the cell-specific activity at each Mediterranean station, this was comparable to those in the literature (e.g., [83] and references therein) and one order of magnitude lower on average than those reported by [79] for the Mediterranean Sea water column at similar depths. This observation suggests that sediment prokaryotes convert organic matter into biomass at a rate comparable to that for other oceans, despite the higher bottom temperature [79].

By contrast, the HCP in the North Atlantic was instead exceptionally high. When the cell-specific activities for this station were calculated, these were 50-fold higher than in any other station. This is not surprising, as the sampled area in the North Atlantic Ocean is positioned on the Galicia Seamount, an upwelling area that is known as highly productive [84,85], and that HCP has been previously positively correlated with primary production in the photic zone [86], probably due to increased top-down mechanisms (e.g., predation and viral lysis). Difference in bottom-water temperatures between sampled stations were expected to positively influence *in-situ* prokaryotic activity [79]. When included in the analysis, bottom temperatures were significant only in separating the North Atlantic stations from the Mediterranean stations, and were inversely correlated with measured HCP (Pearson moment correlation, r = -0.61, n = 60, p <0.001).

One of the major concerns in the direct counting of prokaryotes is the inability to distinguish between active and dormant members of the community [10,51,79]. FISH and CARD-FISH techniques have the power to overcome this issue. By targeting the ribosomes, FISH techniques stain only actively transcribing cells, which leads to the counting of the active fraction of the population [73,87]. Our data show that, on average, a high proportion of total prokaryotes were metabolically active. However, there were big differences in the fractions of active cells between the stations. Despite those local differences there were no significant variations in the fraction of active cells between the sampling areas or the depths (Table 3). Measured differences might be due to variations in the ability to permeabilize the cell membrane in different samples, or more probably to the presence of different fractions of dead or dormant cells [51,61].

On average, the *Bacteria* accounted for *ca* 70% of the total prokaryotes, as previously reported in other studies on surface sediments [9,17,61]. Only at station 17 at 3,000 m in depth in the Central Basin did *Archaea* outnumber *Bacteria*, accounting on average for 53% of the total prokaryotes. Moving eastwards, the BAR decreased significantly, with that in the East Mediterranean Sea lower than those in other areas (Figure 3e). The HCP was significantly correlated with the BAR (Pearson moment correlation, r = 0.531, n = 60, p <0.001), which suggests, on average, a dominance of heterotrophic *Bacteria* in deep-sea surface sediments as recently reported by Molari et al. [48]. The low BAR in the Eastern Basin might be coupled to the marked oligotrophy of the East Mediterranean surface waters (Table 1, SPP), which is reflected in the low availability of organic matter for benthic consumers (Figure 2a, b). This appears especially true when considering the CPE and PRT content as proxies for quality and bioavailability of sedimentary organic matter [81]. On the other hand, the increased importance of *Archaea* might be related to an increased contribution of dark carbon fixation in the area, as already suggested by Yakimov et al. [88]. It is believed that *Crenarchaeota* MG-I drive the dark carbon fixation in deep water that couples CO_2_ fixation to ammonia oxidation (e.g., [89]). Several studies have been published on this [13,88,90,91], and an increase in the abundance of *Crenarchaeota* is considered a proxy for the increased importance of chemolithoautotrophy linked to ammonia oxidation. Recently, *Crenarchaeota* MG-I, of which ammonia oxidizers are part, have been reported in marine sediments [33,61,92] and marine basalts [93], where they are actively involved in inorganic carbon assimilation [48]. Their abundance in the surface sediments is not surprising. Our results show that the contribution of *Crenarchaeota* MG-I to the total pool of *Archaea* is relatively constant across the investigated area (Figure 3e), despite the increased contribution of *Archaea* to the total prokaryotes in the East Mediterranean.

Previous studies have suggested that *Crenarchaeota* typically dominate over *Euryarchaeota* in oxygenated deep waters [22,25] and surface sediments [32,33,61]. We found the same pattern in our survey, with the ECR constant between areas, with values below 1 showing *Crenarchaeota* MG-I domination of the *Archaea* community, except for station 18 (Matapan-Vavilov Deep) where *Euryarchaeota* MG-II accounted for 60% of the *Archaea* population. *Euryarchaeota* MG-II has been previously reported to dominate over *Crenarchaeota* in surface waters [31,60,94]. In particular, Massana et al. [31] concluded that *Euryarchaeota* MG-II are dominant only at the surface in temperate waters. Our data clearly show an increased importance of *Euryarchaeota* at the Matapan-Vavilov Deep (station 18), raising interesting questions as to the role of this yet-uncultured group that requires further investigation. A recently published reconstruction of an MG-II *Euryarchaeota* genome from metagenomic sequences [95] suggests that this group comprises heterotrophs that can grow on lipids and fatty acids. Interestingly station 18 had one of the highest concentrations of lipids in our dataset (Table 2), and the Matapan-Vavilov Deep has been previously reported to be a possible organic matter sink [76] due to its depth and proximity to land masses.

### Effects of trophic and environmental variables on prokaryotic assemblages

Combining all of the prokaryotic variables measured to describe the prokaryotic assemblages as a whole, we found that prokaryotic communities in deep-sea surface sediments share similarities across the entire Mediterranean basin and North Atlantic stations (Figure 4). In particular, the communities from the Central Mediterranean stations are interspersed between all of the other areas (Figure 4a). The prokaryotic communities from the East Mediterranean stations clustered in a single condensed group, which suggests that there are only minor differences between the community at 1,200 m in depth and 3,000 m in depth in this area.

When we separated the analysis based on the depth, there were striking differences between the prokaryotic communities of the North Atlantic compared to the Mediterranean Sea at 1,200 m in depth (Figure 4b). Those differences were not as clear at 3,000 m in depth (Figure 4d), and they were absent at 2,000 m in depth (Figure 4c), where the points were highly interspersed. These data suggest that the deeper areas of different basins share more similar communities with each other than with shallower sites, and that depth is indeed an important variable structuring the prokaryotic assemblages within each area.

We also wanted to identify the trophic and environmental variables that influence the prokaryotic assemblage composition and activity in the surface deep-sea sediments. In energetically stressed environments, such as the deep-sea [96], it is often difficult to identify processes that affect the distribution and abundance of different biotic assemblages. This is because both biological and physical factors are involved, they are not independent, and their relationships with the taxa of interest are frequently non-linear. To identify relationships in such situations the use of most conventional correlational-type approaches is inappropriate [97]. In the present study, we used the non-linear model-building approach of GAMs. This facilitates the description of the relationships between the response variables and each predictor, while simultaneously adjusting for covariation among the predictors. To our knowledge this is the first time that GAMs have been applied to microbial ecology in marine environments.

Trophic variables were identified by both linear regression and GAMs analysis applied to our ordination (Figure 5), as the main forcing variables in the shaping of the prokaryotic assemblage composition differences across the stations. The BPC was effectively related to the ordination by a linear relationship (Figure 5), as can be seen by the presence of equidistant parallel surface lines. The PRT effect on the ordination was non-linear, as was the effect of the LIP concentration. The GAM-nMDS superimposed analysis further identified LONG as an important factor, together with SPP and CPE.

These variables were then used in the GAMs analysis to identify the driving factor that describes the BAR, ECR and HCP variations in our dataset. The resulting models had very powerful prediction efficiencies, as can be seen by plotting the measured against fitted values (Figure 6). Out of the three models, the HCP-GAM model had the highest accuracy (Pearson moment correlation, r = 0.972, p <0.001, n = 60, Figure 6c), with the HCP variance in our dataset explained by a combination of *Bacteria* abundance, trophic variables, and position along the longitudinal gradient. The BAR variations were successfully explained by a combination of geographic and trophic variables. The BAR-GAM model showed good prediction accuracy (Pearson moment correlation, r = 0.948, p <0.001, n = 69; Figure 6a), with all of the variables modeled as non-linear. Our data show that the quality of the organic matter is of primary importance in determining the community BAR. PRT and CPE are good proxies for organic matter quality, as they are readily degradable substrates that tend to disappear as organic matter ages and undergoes burial [81]. The interaction between SPP and CPE increased the model-explained variance by *ca* 10%, as compared to a similar model where the SPP and CPE were considered independently. The amount of CPE in deep-sea sediments has been shown to be a function of the SPP, depth and efficiency of removal along the water column as the organic matter particles sink to the seafloor [81]. It is not a surprise that the two variables had an interaction effect in describing the quality of organic matter in the surface deep-sea sediments.

The ECR-GAM model was strongly influenced by the variations in the ECR ratio at station 18 (Matapan-Vavilov Deep). The depth, together with the BAR and geographic position, had a powerful predictive power (Pearson moment correlation, r = 0.902, p <0.001; n = 69), as can be seen by plotting the measured against the fitted ECR values (Figure 6b).

The effect of longitude and its interactions with latitude in explaining our variance in all three models is an intriguing finding. This implies that geographic position along the Mediterranean Sea has a strong influence on the activity and structure of the prokaryotic assemblages that is not related to any other variables measured in the present study. Previous studies analyzing differences between the West and East Mediterranean basins have shown that the main differentiating features were primary productivity and nutrients in the water column [98], with a more productive area in the West Mediterranean [99], and salinity [100] and minor variations in the bottom temperatures [101,102]. Of these traditionally accounted variables, our dataset includes surface primary production, trophic resources in surface sediments, and temperature. Despite this, the effects of latitude and longitude are still strong, and might hide the effects of a variable or set of variables that were not measured in the present survey. This suggests that overlying water masses might have critical roles in shaping deep-sea benthic prokaryotic assemblage composition.
